# Socioeconomic inequalities in access to specialized psychotropic prescribing among older Swedes: a register-based study

**DOI:** 10.1093/eurpub/cku058

**Published:** 2014-05-24

**Authors:** Jonas W. Wastesson, Johan Fastbom, Gunilla Ringbäck Weitoft, Stefan Fors, Kristina Johnell

**Affiliations:** 1 Aging Research Center, Karolinska Institutet and Stockholm University, Stockholm, Sweden; 2 National Board of Health and Welfare, Stockholm, Sweden

## Abstract

**Background:** Mental disorders among older adults are mainly treated with psychotropic drugs. Few of these drugs are, however, prescribed by specialized geriatricians or psychiatrists, but rather from general practitioners (GPs). Socioeconomic inequalities in access to specialist prescribing have been found in younger age groups. Hence, we aimed to investigate whether there are socioeconomic differences in access to geriatrician and psychiatrist prescribing of psychotropic drugs among older adults. **Methods:** By record-linkage of The Swedish Prescribed Drug Register and The Swedish Education Register, we obtained information for persons aged 75–89 years who had filled a prescription for psychotropic drugs (antipsychotics, anxiolytics, hypnotic/sedatives or antidepressants) with information on prescriber specialty from July to October 2005 (*n* = 221 579). Multinomial regression analysis was used to investigate whether education was associated with geriatrician and psychiatrist prescribing of psychotropic drugs. **Results:** The vast majority of the psychotropic drugs were prescribed by ‘GPs and other specialists’ (∼95% GPs). Older adults with higher educational level were more likely to be prescribed psychotropic drugs from psychiatrists and geriatricians. However, after adjustment for place of residence, the association between patient’s education and prescription by a geriatrician disappeared, whereas the association with seeing a psychiatrist was only attenuated. **Conclusion:** Access to specialized prescribing of psychotropics is unequally distributed between socioeconomic groups of older adults in Sweden. This finding was partially confounded by place of residence for geriatrician but not for psychiatrist prescribing. Further research should examine if inequalities in specialized psychotropic prescribing translate into worse outcomes for socioeconomically deprived and non-metropolitan-living older individuals.

## Introduction

Mental disorders are common among older adults. It is estimated that about one in four are afflicted by at least one mental disorder^1^ or by psychological distress^2^ in the elderly population. This will pose an increasing public health problem as more people reach old age.^3^ Mental disorders among older adults are mainly treated with pharmaceuticals. Indeed, psychotropic drugs are among the most commonly used drugs among older adults.^4^ In Sweden, general practitioners (GPs) prescribe the majority of all psychotropic drugs to older adults, whereas specialized geriatricians and psychiatrists only prescribe a small share, about 10 and 5%, respectively.^5^

Prescribing of psychotropics to older patients is complicated by the altered pharmacokinetics and pharmacodynamics of the ageing body, which has implications for the choice of drug, the dose and the monitoring of side effects.^6^ The high rates of comorbidity and polypharmacy among older adults further complicate the practice of prescribing to older persons.^7^ Moreover, symptoms of mental disorders may appear differently among older and younger patients. This may, in turn, cause misdiagnosis and mistreatment of elderly persons.^8^ Therefore, access to a geriatrician or a psychiatrist may be beneficial for older persons.^9–11^ A licensed physician without a specialty in Sweden only undergoes about 2 weeks of training in geriatric medicine.^12^ In addition, there is a shortage of geriatric psychiatrists in Sweden^13^ as well as in other countries.^14^ Thus, older adults in Sweden have limited access to specialized care for mental disorders.^5^

Socioeconomic inequalities in the utilization of specialist services have been found in many countries and for a wide range of diagnoses.^15–17^ In general, most countries show a pattern of no or small inequalities in the utilization of GPs in relation to need. However, the use of specialist services tends to have a ‘pro-rich bias’, where affluent persons are more likely to receive specialist care.^15–17^ Most studies have focused on overall use of health care, which might obscure socioeconomic inequalities in different types of health care services and in certain patient groups.^18^ Relatively few studies have focused on older adults or on the provision of drug treatment.^18^^,^^19^ In one of the few studies of specialist prescribing, neurologists rather than GPs were shown to more often be the prescriber of antiepileptic drugs if the patient was younger, highly educated, a high-income earner or lived in a large city.^20^ Specialist prescribing of psychotropic drugs to older adults has, to our knowledge, not previously been analysed in terms of inequality. Older persons with mental disorders should have equal access to geriatricians and psychiatrists regardless of their socioeconomic conditions.

Thus, the main objective of this study is to investigate whether there are socioeconomic differences in access to geriatrician and psychiatrist prescribing of psychotropic drugs among older adults in Sweden. A second aim is to investigate if geographical variations contribute to these potential socioeconomic differences.

## Methods

### Study population

The Swedish Prescribed Drug Register (SPDR) contains detailed data on prescribed and dispensed drugs for the whole population of Sweden. Data are collected from Swedish pharmacies and kept by the Swedish National Board of Health and Welfare.^21^ In this study, we analysed individual-based data on prescribed drugs dispensed from July to October 2005. As the maximum treatment duration for a single prescription in Sweden is 3 months, all regularly used drugs should be covered by the 4-month study period. The dispensed drugs were classified according to the Anatomical Therapeutic and Chemical classification system (ATC), as recommended by the World Health Organization.^22^

Individual prescribers cannot be identified in the SPDR, but some key prescriber characteristics for each drug are included, such as prescriber profession (nurse/dentist/physician) and physician speciality (62 different specialities available in Sweden).^5^ Because we were only interested in physicians and their specialties, we excluded all drugs prescribed by other professionals.

Data from the SPDR were record-linked to The Swedish Education Register.^23^ This register contains information on the highest attained level of formal education for Swedish citizens aged 16–74. By collecting information from the years 1990, 1997, 2000 and 2004, we were able to obtain data on education from people as old as 89 years in 2005.

### Sample

Among persons aged 75–89 years, 660 086 had filled a prescription during the study period and were included in the SPDR. This register covers around 85–90% of the total population of Swedes in these age groups.^4^ From the cohort, we then extracted those individuals who had filled a prescription for at least one psychotropic drug:^24^ antipsychotics (ATC code N05A), anxiolytics (N05B), hypnotic/sedatives (N05C) or antidepressants (N06A) (*n* = 246 473). Of these, 8% (*n* = 19 199) were excluded owing to lack of information on prescriber’s speciality on all drug types under study, 2% (*n* = 5624) owing to missing information on education and <1% (*n* = 71) owing to missing information on place of residence. The final study population consisted of 221 579 individuals.

### Measurements

Specialized prescribing was divided into the categories ‘geriatrician’, ‘psychiatrist’ and ‘GPs and other specialists’. GPs are defined as specialists in family medicine in the Swedish system and therefore included in the category ‘GPs and other specialists’. The vast majority of the individuals with prescriptions from ‘GPs and other specialists’ were prescribed psychotropics by a GP (about 95%). If a person was prescribed a drug from both ‘geriatrician’/‘psychiatrist’ and ‘GPs and other specialist’, the person was coded as having been prescribed a drug from a ‘geriatrician’/‘psychiatrist’. Few persons, 0.4% (*n* = 807), were prescribed psychotropics from both a geriatrician and a psychiatrist and were therefore too few to be analysed separately. Because these persons were few, the estimates did not change depending on whether they were coded as filling prescriptions by a psychiatrist or a geriatrician. In the final model, they were coded as having seen a psychiatrist, because fewer persons saw a psychiatrist and persons referred to psychiatrists are probably a more selected group.

Educational level was coded as low education (<9 years of education), medium education (9–12 years of education) and high education (>12 years of education).^25^

Place of residence was categorized into ‘metropolitan areas’ and ‘non-metropolitan areas’. Metropolitan areas included the three largest cities in Sweden (i.e. Stockholm, Gothenburg and Malmoe) and non-metropolitan areas were all other places. Dementia status was assessed by filling a prescription for an anti-dementia drug (ATC code N06D) and was coded as yes/no.

Age and number of drugs used, which has been shown to be a valid proxy measure for overall comorbidity,^26^ were included in the analyses as continuous variables.

### Statistical analysis

The association between a person’s educational level and being prescribed psychotropic drugs from geriatricians and psychiatrists compared with ‘GPs and other specialists’ was assessed by bivariate and multivariate (adjusted for age, gender, place of residence, dementia status and number of drugs) multinomial regression models. The analyses were performed for the four types of psychotropics separately (i.e. antipsychotics, anxiolytics, hypnotics/sedatives and antidepressants). Results are presented as relative risk ratios with 95% confidence intervals. Persons with a psychotropic prescription from the category ‘GPs and other specialists’ were considered as the reference category in all analyses.

To account for the geographical clustering of individuals, over and above living in a metropolitan or non-metropolitan area, the Huber–White sandwich estimator was used in all the regression analyses. The persons were clustered according to municipalities (*n* = 290), which are the smallest local government entities in Sweden. This was done to account for unobserved characteristics at the municipality level. STATA 11 was used for the analyses.

## Results

Of the 221 579 elderly psychotropic users, 87% (*n* = 192 324) used only one of the studied psychotropics, 9% (19 741) used two psychotropics, 4% (8707) used three psychotropics and less than 1% (*n* = 807) used all four different types of psychotropics. Of the study population, 9% had received specialized prescribing from a geriatrician and 4% from a psychiatrist (table 1).

**Table 1:** Characteristics of the study population according to use of psychotropic drugs in Sweden, July to October 2005 (*n* = 221 579)

Characteristics	Total study population (*n* = 221 579)	Antipsychotics (*n* = 26 084)	Anxiolytics (*n* = 70 842)	Hypnotics/sedatives (*n* = 139 797)	Antidepressants (*n* = 90 991)
	% (*n*)	% (*n*)	% (*n*)	% (*n*)	% (*n*)
Age, median (IQR)	82 (78–85)	82 (79–85)	82 (78–85)	82 (78–85)	82 (78–85)
Women	69.5 (153 914)	67.3 (17 550)	72.1 (51 078)	69.5 (97 227)	71.6 (65 122)
Education					
Low	56.5 (125 212)	63.2 (16 476)	59.7 (42 300)	54.7 (76 407)	58.1 (52 904)
Medium	28.1 (62 323)	25.0 (6522)	27.2 (19 290)	29.0 (40 525)	27.7 (25 205)
High	15.4 (34 044)	11.8 (3086)	13.1 (9252)	16.4 (22 865)	14.2 (12 882)
Prescribing physician					
Geriatrician	8.9 (19 741)	9.9 (2591)	6.0 (4257)	5.3 (7429)	7.3 (6639)
Psychiatrist	3.9 (8707)	9.3 (2412)	4.4 (3129)	2.5 (3476)	4.7 (4257)
Geriatrician and psychiatrist	0.4 (807)	0.2 (55)	0.2 (117)	0.1 (104)	0.2 (151)
GPs and other specialists	86.8 (192 324)	69.7 (18 191)	80.6 (57 077)	83.2 (116 381)	77.7 (70 713)
Not specified	0	10.9 (2835)	8.8 (6262)	8.9 (12 407)	10.1 (9231)
Metropolitan areas	30.2 (66 899)	27.9 (7279)	30.5 (21 627)	31.4 (43 945)	29.1 (26 449)
Dementia	5.1 (11 374)	11.6 (3016)	5.0 (3546)	3.7 (5177)	8.4 (7652)
Number of drugs, median (IQR)	8 (5–11)	9 (6–12)	9 (6–12)	8 (5–11)	9 (6–12)

IQR: interquartile range.

‘GPs and other specialists’ (∼95% GPs) prescribed the majority of all psychotropic drugs. Antipsychotics were more often prescribed by geriatricians and psychiatrists compared with the other psychotropics. The majority (about 70%) of the psychotropic users were women. In the study population, 5% were treated with anti-dementia drugs and use of these drugs was more common among antipsychotic and antidepressant users.

‘GPs and other specialists’ prescribed the majority of all psychotropic drugs across all educational groups (63–83%) (table 2). The proportion of drugs prescribed from other specialists was largest for hypnotics/sedatives and lowest for antipsychotics. For the four different psychotropic drug types, specialized prescribing from geriatricians and psychiatrists was more common among persons with high education than medium and low education (*P* < 0.05).

**Table 2:** Frequency of specialist prescribing according to educational level among psychotropic drug users 75–89 years of age in Sweden, July to October 2005

Prescriber speciality	Low education %	Medium education %	High education %	*P*-value^a^
**Antipsychotics (*n* = 26 084)**	16 476	6522	3086	
Prescribing physician				
Geriatrician	9.0	11.3	12.1	
Psychiatrist	7.9	10.4	13.8	
Geriatrician and psychiatrist	0.2	0.2	0.1	
GPs and other specialists	72.1	67.2	62.6	
Not specified	10.8	10.8	11.3	<0.001
**Anxiolytics (*n* = 70 842)**	42 300	19 290	9252	
Prescribing physician				
Geriatrician	5.6	6.7	6.6	
Psychiatrist	3.6	4.9	7.0	
Geriatrician and psychiatrist	0.1	0.2	0.2	
GPs and other specialists	81.7	79.6	77.5	
Not specified	9.0	8.6	8.7	<0.001
**Hypnotics/sedatives (*n* = 139 797)**	76 407	40 525	22 865	
Prescribing physician				
Geriatrician	5.1	5.4	5.8	
Psychiatrist	2.1	2.6	3.5	
Geriatrician and psychiatrist	0.1	0.1	0.1	
GPs and other specialists	83.3	83.4	82.9	
Not specified	9.4	8.5	7.7	<0.001
**Antidepressants (*n* = 90 991)**	52 904	25 205	12 882	
Prescribing physician				
Geriatrician	6.8	7.9	8.1	
Psychiatrist	3.7	5.2	7.6	
Geriatrician and psychiatrist	0.1	0.2	0.2	
GPs and other specialists	79.0	76.9	74.1	
Not specified	10.4	9.8	10.0	<0.001

a: *P*-values calculated with χ^2^ test.

Table 3 displays the association between the patient’s educational level and prescriber speciality (unadjusted Model I), after controlling for age, gender, dementia status and overall comorbidity (partially adjusted Model II) and after additional adjustment for place of residence (fully adjusted Model III) for the four types of psychotropics separately. For all of the psychotropics (antipsychotics, anxiolytics, hypnotics/sedatives and antidepressants), having a higher educational level was associated with being prescribed by a geriatrician or psychiatrist in comparison with ‘GPs and other specialists’ in the unadjusted model (Model I) and in the partially adjusted model (Model II). When place of residence was entered into the model (Model III), the associations between patient’s educational level and the odds of being prescribed by a geriatrician became statistically non-significant and the associations between education and the odds of being prescribed by a psychiatrist were attenuated.

**Table 3:** Relative risk ratios (RRRs) with 95% confidence intervals (95% CIs) of psychotropic prescribing by a geriatrician or a psychiatrist compared with prescribing by GPs and other specialists among psychotropic drug users 75–89 years of age in Sweden, July to October 2005

Variables	Geriatrician			Psychiatrist		
	Model I	Model II	Model III	Model I	Model II	Model III
	RRR (95% CI)	RRR (95% CI)	RRR (95% CI)	RRR (95% CI)	RRR (95% CI)	RRR (95% CI)
**Antipsychotics (*n* = 23 249)**						
Education						
Low	1	1	1	1	1	1
Medium	1.35 (1.19–1.54)	1.33 (1.16–1.52)	1.06 (0.93–1.20)	1.42 (1.27–1.58)	1.35 (1.20–1.52)	1.26 (1.14–1.39)
High	1.55 (1.35–1.79)	1.56 (1.35–1.81)	1.11 (0.91–1.36)	1.98 (1.68–2.32)	1.86 (1.60–2.16)	1.66 (1.46–1.89)
Age		1.01 (1.00–1.03)	1.01 (1.00–1.02)		0.86 (0.85–0.87)	0.86 (0.85–0.87)
Women		1.06 (0.97–1.15)	0.99 (0.89–1.10)		1.18 (1.03–1.34)	1.15 (1.00–1.32)
Dementia		1.85 (1.62–2.11)	1.77 (1.55–2.03)		0.46 (0.33–0.64)	0.45 (0.33–0.62)
Number of other drugs		1.03 (1.02–1.04)	1.02 (1.01–1.03)		0.95 (0.93–0.96)	0.94 (0.93–0.96)
Metropolitan areas			4.50 (2.99–6.76)			1.73 (1.40–2.13)
**Anxiolytics (*n* = 64 580)**						
Education						
Low	1	1	1	1	1	1
Medium	1.22 (1.11–1.35)	1.24 (1.12–1.37)	1.07 (0.99–1.15)	1.38 (1.23–1.54)	1.31 (1.17–1.47)	1.25 (1.13–1.37)
High	1.25 (1.10–1.41)	1.28 (1.12–1.45)	1.00 (0.88–1.15)	1.98 (1.69–2.31)	1.84 (1.56–2.18)	1.70 (1.48–1.95)
Age		1.04 (1.02–1.06)	1.04 (1.02–1.05)		0.90 (0.89–0.91)	0.90 (0.89–0.91)
Women		0.96 (0.90–1.04)	0.92 (0.85–0.99)		1.04 (0.96–1.14)	1.03 (0.94–1.12)
Dementia		2.76 (2.41–3.16)	2.83 (2.47–3.25)		1.00 (0.78–1.28)	1.01 (0.78–1.30)
Number of other drugs		1.05 (1.04–1.06)	1.05 (1.04–1.06)		1.01 (1.00–1.02)	1.01 (1.00–1.02)
Metropolitan areas			2.85 (2.10–3.86)			1.47 (1.17–1.84)
**Hypnotics/sedatives (*n* = 127 390)**						
Education						
Low	1	1	1	1	1	1
Medium	1.03 (0.95–1.13)	1.09 (1.00–1.19)	0.95 (0.88–1.02)	1.24 (1.11–1.39)	1.19 (1.06–1.33)	1.12 (1.02–1.22)
High	1.12 (1.01–1.24)	1.18 (1.05–1.31)	0.94 (0.83–1.07)	1.64 (1.44–1.88)	1.43 (1.26–1.61)	1.43 (1.08–1.89)
Age		1.04 (1.03–1.05)	1.04 (1.03–1.05)		0.91 (0.90–0.92)	0.91 (0.90–0.92)
Women		0.90 (0.84–0.95)	0.86 (0.81–0.92)		1.09 (1.02–1.17)	1.07 (1.00–1.15)
Dementia		3.10 (2.71–3.56)	3.17 (2.76–3.63)		1.59 (1.26–2.01)	1.60 (1.27–2.03)
Number of other drugs		1.06 (1.05–1.07)	1.06 (1.05–1.07)		1.03 (1.03–1.04)	1.03 (1.03–1.04)
Metropolitan areas			2.48 (1.87–3.29)			1.52 (1.18–1.96)
**Antidepressants (*n* = 81 760)**						
Education						
Low	1	1	1	1	1	1
Medium	1.20 (1.10–1.30)	1.22 (1.12–1.33)	1.05 (0.97–1.13)	1.44 (1.29–1.60)	1.36 (1.22–1.52)	1.27 (1.17–1.39)
High	1.27 (1.11–1.45)	1.27 (1.11–1.45)	0.98 (0.85–1.13)	2.14 (1.91–2.39)	1.95 (1.30–2.44)	1.73 (1.56–1.92)
Age		1.03 (1.01–1.05)	1.03 (1.01–1.05)		0.91 (0.90–0.91)	0.90 (0.90–0.91)
Women		0.90 (0.85–0.97)	0.86 (0.80–0.91)		1.02 (0.95–1.10)	1.00 (0.93–1.08)
Dementia		2.51 (2.19–2.86)	2.55 (2.21–2.94)		0.97 (0.74–1.27)	0.98 (0.75–1.29)
Number of other drugs		1.04 (1.03–1.05)	1.03 (1.03–1.04)		0.99 (0.98–1.00)	0.99 (0.98–1.00)
Metropolitan areas			3.02 (2.20–4.14)			1.72 (1.36–2.18)

Adjustment for potential confounders, except for place of residence, only had modest effects on the estimates of education. Place of residence attenuated the associations in all analyses, particularly for seeing a geriatrician. People residing in metropolitan areas had a 2–4 times higher likelihood of seeing a geriatrician than persons living in non-metropolitan areas, compared with a 1.5–2 times larger likelihood of seeing a psychiatrist. Further, dementia was positively associated with seeing a geriatrician in all analyses, but not with seeing a psychiatrist. Older age was positively related to seeing a geriatrician and inversely related to seeing a psychiatrist.

Interaction terms between gender and education and metropolitan-living and education were significant in the full models but did not modify the association between education and prescriber speciality. However, among anxiolytic drug users, the association between education and being prescribed by a psychiatrist was no longer statistically significant among women (odds ratio_low __education vs. high education_: 1.12; 95% confidence interval: 0.92–1.38).

## Discussion

In this nationwide study, we found that among older persons who used psychotropics, higher education was associated with prescriptions from a psychiatrist or a geriatrician rather than from ‘GPs and other specialists’ (∼95% GPs). Higher educational level was associated with being prescribed from psychiatrists or geriatricians for all psychotropic drug types. The associations were, however, attenuated after adjustment for place of residence, particularly for seeing a geriatrician. Older people who lived in metropolitan areas were much more likely to receive specialized psychotropic prescribing than their non-metropolitan-living counterparts.

There has been a growing awareness of the problems linked to treating older adults with psychotropics^27^ and the inappropriate use of these drugs.^28^ This implies complex challenges for the physician that sometimes require specialist geriatric or psychiatric competence. As a consequence, the current situation in Sweden where older adults with mental disorders have limited access to geriatricians and psychiatrists has been criticized^5^ because other physicians may not receive enough training in geriatric psychiatry.^12^ In a previous register-based study of Swedes aged 65 years and older, GPs were the main prescribers of psychotropic drugs and only 10% had been prescribed psychotropics from a geriatrician and 5% from a psychiatrist,^5^ which is in line with the results from this study. Regardless, access to specialist prescribing should not be dependent on the person’s socioeconomic conditions.

Most studies of socioeconomic inequalities in utilization of specialist services are general population studies.^15^ Few have focused specifically on older adults^18^ or on mental disorders.^20^ Our findings of socioeconomic inequalities in prescribing are in line with a previous study from Sweden about inequalities in antiepileptic drug prescriptions to adult epilepsy patients.^20^ That study also found that lower socioeconomic position was associated with less specialized prescribing (neurologist); the same pattern that we found in our study for psychiatrist prescribing. Apart from the previous Swedish study, research about inequalities in specialist prescribing has been scarce.

We show that persons with higher education were more likely than those with low education to be prescribed psychotropic drugs by a physician who was specialized in older people or mental disorders. The mechanisms leading up to socioeconomic differences in prescribing are likely to be complex and multifaceted.^29^ Sweden has quite a low level of co-payment for drugs,^30^ and very few older adults state that they have refrained from seeking medical care owing to financial reasons.^25^ Thus, affordability is not likely to entirely explain our results. Furthermore, affordability should foremost be an inhibitive factor for taking the active decision to seek care in the first place, and in our study, all included persons had already sought and received some kind of treatment.

However, it is possible that the person’s behaviour when interacting with the health care system could contribute to the results. It has previously been argued that the members of high-income groups are better at articulating their demands and are more able to orient themselves within a complex health care system.^15^ In the Swedish health care system, where the GPs often act as gatekeepers to specialized care, highly educated individuals may have higher referral rates to specialized care. Factors related to the health care system itself can also be a possible explanation. For example, it has been shown that GPs are more likely to take patient demands under consideration if the patient is highly educated, which could also lead to differences in GP referrals.^31^^,^^32^

Geographical distance and transportation are likely important factors for health care utilization, which may, in turn, lead to socioeconomic inequalities in utilization.^33^ The socioeconomic differences in prescribing were partly confounded by the person’s place of residence, as highly educated tended to live in metropolitan areas. After adjustment for place of residence, the association between patients’ education and geriatrician prescribing turned statistically non-significant. However, the association for psychiatrist prescribing was still statistically significant, albeit attenuated. Regional differences in use of mental health services have previously been found in both Sweden^20^^,^^34^ and Finland.^33^ In rural and more remote areas, access to specialized care is limited by long distances. In the present study, older adults residing in metropolitan areas were more likely to receive their prescriptions from geriatricians and psychiatrists rather than GPs. Surprisingly, access to geriatricians was more related to residing in metropolitan areas than access to psychiatrists. This could either indicate that geriatricians are more concentrated in metropolitan areas or that proximity and accessibility are key factors for frail elderly persons when seeing geriatricians.

Dementia status was associated with being prescribed by a geriatrician, indicating that patients with dementia are more likely to receive adequate specialized care.^11^ Older patients were also more likely to be prescribed by a geriatrician. However, for seeing a psychiatrist, lower age was a determinant, which is in line with previous research showing that mental disorders among the oldest-old are a neglected problem.^13^

### Limitations

Only persons with a prescription from physicians with a speciality recorded in the SPDR were included in the study. Prescribers without a speciality are in most cases physicians who have not yet specialized. About 8% of the sample had only filled prescriptions from physicians without a speciality and were therefore excluded. Because higher socioeconomic position is often related to use of specialist services,^15^ exclusion of this group would most likely lead to an underestimation of the association.

Referrals to psychiatrists are probably influenced by the severity of the mental health problems. However, there is no information on indication or severity of disease in the SPDR. Thus, our results could hypothetically be explained by more severe mental health problems among the highly educated. However, there is little support in the literature for this scenario.^35^ In fact, older adults with low socioeconomic position are more likely to concurrently use several psychotropics in Sweden.^36^^,^^37^

Further, as this study is cross-sectional, it is inherent that many of the dispensed prescriptions are refills. Often, the physician who prescribes the refill is not the same as the one who initiated the treatment. Therefore, the prevalence of specialist prescribing should be interpreted with caution. This could also in part explain the association between education and access to geriatricians and psychiatrists if people with higher education were more likely to receive their refills from the physician who initiated the treatment. However, receiving regular treatment from a specialist would also be a potential inequality.

Further, filling a prescription for a psychotropic drug does not necessarily indicate occurrence of a mental disorder. First, some psychotropics can also be prescribed for somatic problems, such as pain.^38^ Second, as the study is cross-sectional, we cannot separate between short- term and long-term use. Thus, our results should not be interpreted as findings about the association between socioeconomic position and mental health problems.

The studied age cohort’s educational attainment was in general low. Thus, including other measures of socioeconomic position (occupational class and/or income) would have allowed for a more refined analysis of the association between socioeconomic position and access to specialized prescribing. However, education is highly associated to other measures of socioeconomic position.^39^ Moreover, we adjusted the analyses for metropolitan/non-metropolitan differences and persons were also clustered within municipalities. However, geographical differences in access to specialized prescribing cannot fully be accounted for in this study and should ultimately be studied within a multi-level framework.

In this large register-based study, we found that highly educated individuals were more likely to be prescribed psychotropics by geriatricians and psychiatrists rather than GPs among older adults in Sweden. For geriatrician prescribing, most of the socioeconomic differences were explained by patients’ metropolitan/non-metropolitan setting. However, for psychiatrist prescribing, geographical setting could not explain the educational differences found. Further research should examine if inequalities in specialized psychotropic prescribing translate into worse outcomes for socioeconomically deprived and non-metropolitan-living older individuals. Equal access to specialist prescribing should be highlighted in health policy.

## Funding

This work was supported financially by the Swedish Research Council [grant number 2012-1503] and the Swedish Research Council for Health, Working Life and Welfare [grant number 2012-0761].

*Conflicts of interest*: None declared.

**Key points**

- Older adults with mental disorders are mainly treated with psychotropics prescribed by GPs.
- Older patients with lower education and who live in non-metropolitan areas are less likely to receive their prescriptions from geriatricians or psychiatrists.
- Older adults with mental disorders should have equal access to geriatricians and psychiatrists regardless of their socioeconomic position and efforts should be made to reach patients in non-metropolitan areas.
- From a policy perspective, there is a need to promote equal access to specialist prescribing from geriatricians and psychiatrists across regions and socioeconomic groups.

*Ethics approval:* This study was approved by the ethical board in Stockholm (Dnr 2006/948-31) and the authors only analysed non-identifiable data.
